# Heat Shock Factor Is Involved in Regulating the Transcriptional Expression of Two Potential Hsps (*AhHsp70* and *AhsHsp21*) and Its Role in Heat Shock Response of *Agasicles hygrophila*

**DOI:** 10.3389/fphys.2020.562204

**Published:** 2020-09-15

**Authors:** Jisu Jin, Youzhi Li, Zhongshi Zhou, Hong Zhang, Jianying Guo, Fanghao Wan

**Affiliations:** ^1^College of Plant Protection, Hunan Agricultural University, Changsha, China; ^2^State Key Laboratory for Biology of Plant Diseases and Insect Pests, Institute of Plant Protection, Chinese Academy of Agricultural Sciences, Beijing, China

**Keywords:** *Agasicles hygrophila*, heat shock protein, heat shock factor, RNAi, thermotolerance

## Abstract

Heat shock proteins are molecular chaperones that are involved in numerous normal cellular processes and stress responses, and heat shock factors are transcriptional activators of heat shock proteins. Heat shock factors and heat shock proteins are coordinated in various biological processes. The regulatory function of heat shock factors in the expression of genes encoding heat shock proteins (Hsps) has been documented in some model insects, however, the role of transcription factors in modulating Hsps in other insects is still limited. In this study, one heat shock factor gene (*AhHsf*) was isolated and its two potential target genes (*AhHsp70* and *AhsHsp21*) were confirmed from *Agasicles hygrophila*. *AhHsf* sequence analysis indicated that it belongs to the Hsfs gene family. RT-qPCR showed that expression levels of heat shock factors and of two heat shock proteins significantly increased under heat stress. Injection with double-stranded *Hsf* RNA in freshly emerged adult beetles significantly inhibited expression of *AhHsp70* and *AhsHsp21*, shortened the adult survival, drastically reduced egg production, and ultimately led to a decrease in fecundity. RNA interference (RNAi)-mediated suppression of *AhHsp70* or *AhsHsp21* expression also significantly affected expression of *AhHsf*. Our findings revealed a potential transcriptional function of AhHsf to regulate expression of *AhHsp70* and *AhsHsp21*, which may play a key role in *A. hygrophila* thermotolerance. Our results improve our understanding of the molecular mechanisms of the AhHsf - AhHsps signaling pathway in *A. hygrophila.*

## Introduction

Organisms respond to elevated temperatures and to several chemical and physiological stressors by increasing the synthesis of heat shock proteins (Hsp) (Wu, 1995; Tatar et al., 1997; Feder and Hofmann, 1999; Kristensen et al., 2003; Sørensen et al., 2003). Inducible expression of heat shock genes is a response to a plethora of stress signals (Lis and Wu, 1993; Morimoto, 1993; Wu, 1995) that are triggered by (1) abiotic stressors such as irradiation, temperature, salinity, and drought and (2) biotic stressors such as natural enemies and pathogen invasion (Lindquist and Craig, 1988). The heat shock response was first described by Ritossa (1962) who observed an induction of specific chromosome puffs on the polytene chromosomes in *Drosophila melanogaster* following heat or chemical treatment; this constitutes an efficient defense system against detrimental effects of protein denaturation that are predominantly associated with heat stress. Subsequent studies elucidated the nature of induced RNAs and proteins at a molecular level and led to the isolation and characterization of *Hsp* genes (Lindquist, 1986; Lindquist and Craig, 1988; Nover, 1991; Lü et al., 2014; Deng et al., 2018; Jin et al., 2020a). Central to the heat shock response is the induction of Hsps that effectively counteract the effects of stress by stabilizing, re-folding, or degrading denatured proteins (Parsell and Lindquist, 1993).

Stress-induced transcription requires activation of a heat shock factor protein (Hsf) (Lis and Wu, 1993; Morimoto, 1993; Wu, 1995; Voellmy, 2004) that binds to the heat shock promoter element (HSE) (Pelham, 1982). The heat shock response element is composed of three contiguous inverted repeats of a 5-base-pair (bp) sequence whose consensus was defined as nGAAn (Amin et al., 1988; Xiao and Lis, 1988) and was recently revised to AGAAn (Cunniff and Morgan, 1993; Fernandes et al., 1994; Kroeger and Morimoto, 1994). Hsf is present in a latent state under normal conditions and typically occurs as an inactive protein (Zhong et al., 1996), however, Hsf is activated in response to heat stress (Nair et al., 1996; Voellmy, 2004; Marchler and Wu, 2014), and activation of Hsf generally occurs in three stages: (a) thermally induced formation of homologous trimers and hyperphosphorylation, (b) transfer to the nucleus and recognition, and (c) binding to HSE domain sequences of specific protein genes (Sarge et al., 1993; Morimoto, 1993; Jolly et al., 1999; Cohen, 2000; Scharf et al., 2012). Sequencing of *Hsf* genes of numerous species has provided crucial information for understanding the heat shock signaling pathway (Schuetz et al., 1991; Wisniewski et al., 1996; Gonsalves et al., 2011). A considerable amount of studies has been conducted on how Hsfs are changed in response to heat stress, however, the mechanism by which these particular stress signals are transduced to *Hsf* genes remain unclear. Previous studies observed up-regulation of the gene *Hsp70* in response to long-term cold exposure, indicating activation of Hsfs (Burton et al., 1988; Goto and Kimura, 1998; Sejerkilde et al., 2003), and Burton et al. (1988) observed increased survival of *D. melanogaster* larvae receiving a mild heat treatment prior to cold stress, suggesting cross-resistance to different stressors.

Stress response mechanisms following high temperature knock down (Huey et al., 1992) are likely to be closely associated with heat stress responses, thus it is reasonable to speculate that Hsps may exert a protective effect. Previous studies showed that expression of heat shock proteins increases survival in *D. melanogaster* (McColl et al., 1996).

Alligator weed [*Alternanthera philoxeroides* (Mart.) Griesb. (Amaranthaceae)] originates from South America and has become highly invasive in numerous countries in North and Central America, Australia, and Asia (Julien et al., 1995; Buckingham, 1996; Xu et al., 2003; Zhang et al., 2016). In China, this species was first introduced as a forage crop in the late 1930s, and it subsequently spread to the country’s southern regions (Wang et al., 2005; Ye et al., 2010). *A. philoxeroides* is a well-adapted species, and it can reproduce both sexually and asexually, although the seeds are often not viable (Julien et al., 1995). It causes economic and ecological problems in almost all its newly invaded habitats (Maddox and Rhyne, 1975). The flea beetle *Agasicles hygrophila* Selman & Vogt (Coleoptera: Chrysomelidae) has shown to be an efficient biological control agent of *A. philoxeroides* after investigations by the United States Department of Agriculture in South America from 1960 to 1974 (Zeiger, 1967; Spencer and Coulson, 1976; Coulson, 1977; Buckingham, 1996; Zhao et al., 2016). *A. hygrophila* was first introduced in China in 1986, and it effectively helped control *A. philoxeroides* spreading in the southern regions of China (Wang, 1989; Pan et al., 2006). This was also the most successful case of biological control of exotic weeds in China (Ma et al., 2003). However, field surveys showed that the population density of *A. hygrophila* typically decreases markedly from July to September during midsummer in Hunan province, China, leading reduced inhibition of *A. philoxeroides* growth (Li et al., 2011). In addition, our analysis of temperature data from 2003 to 2013 in Changsha City showed that in May and June, the frequency of high ambient temperatures over 36°C was only 0.29 and 3.3%, respectively. In July and August, the frequencies of temperatures over 36°C were 42.5 and 32.1%, respectively; the daily maximum temperatures often exceeded 39°C. In September, the frequency of high temperatures over 36°C was still high [Jin et al., 2020b (Accepted); Supplementary Table S1].

Previous studies reveal that the three *Hsp* genes play important roles for thermotolerance in *A. hygrophila* (Jin et al., 2020a), however, the respective molecular mechanisms affecting thermotolerance are poorly understood. In the present study, we identified a heat shock factor (AhHsf) and two of its potential downstream target genes, *AhHsp70* and *AhsHsp21* via gene functional verification. Reverse-transcription quantitative polymerase chain reactions (RT-qPCR) and RNA interference (RNAi) techniques were used to assess the effect of *Hsf* on expression of two *Hsp* genes and fecundity of *A. hygrophila.* Our study helps improve the understanding of the mechanisms of thermotolerance in *A. hygrophila* at a molecular level, understand the adaptability of *A. hygrophila* to temperature changes, and predict the efficacy of bio-control using *A. hygrophila* in the face of climate change.

## Materials and Methods

### Host Plants and Experimental Insects

Rhizomes and roots of *A. philoxeroides* were collected from a pond at the Institute of Plant Protection, Hunan Academy of Agricultural Sciences, China, and were planted in plastic vessels (30 × 30 × 30 cm) containing sterilized soil. *A. philoxeroides* plants were placed in a greenhouse of the Langfang Experimental Station, Chinese Academy of Agricultural Sciences, Hebei province, China, and were watered daily.

Adult specimens of *A. hygrophila* were collected in June 2017 from a field in Changsha (28°11′49″N, 112°58′42″E), Hunan province, China. Numerous specimens were collected using the sweeping method and were then applied to the experimental plants in the laboratory at the Chinese Academy of Agricultural Sciences, Beijing, China. Plants were grown at a temperature of 26 ± 1°C, 80 ± 5% relative humidity, and under a photoperiod of 12:12 h light:darkness (Guo et al., 2011). The gender of the experimental insects was determined by the presence of a groove at the end of the abdomen (< 12 h after eclosing); this groove was present in males and absent in females (Supplementary Figure S1). Groups of five females and five males were each placed in a cylindrical box of 8 cm diameter and 12 cm height which contained fresh *A. philoxeroides* stems.

### Sample Collection

Newly hatched (<12 h) adult *A. hygrophila* specimens were used in the experiments. Each group of five pairs was placed in one cylindrical box as detailed above which was then covered with gauze. The effects at each temperature was studied using 10 boxes of adult *A. hygrophila* beetles. The temperature tolerance test was set as described in Table 1. Insects were exposed to 30, 36, or 39°C for 4 h (10:00 a.m. to 2:00 p.m.) each day using a constant temperature incubator (PRX-450D-30, Saifu, China). A control group was exposed to 30°C. To analyze the expression of *AhHsp70* and *AhsHsp21*, five females and five males were collected from each temperature group every day for a week. A total of 50 pairs of adults for each temperature treatment were used and three replicates were performed for each treatment. The sampled individuals were immediately frozen in liquid nitrogen and subsequently stored in a −80°C-freezer (DW-86L628, Haier special electric appliance co. Ltd., Tsingtao) until further analyses.

**TABLE 1:** Temperature tolerance test setting of the *A. hygrophila* in this experiment.

**Time of day (hours)**	**Treatment temperature (°C)**		
	**Control**	**Treatment (1)**	**Treatment (2)**
00:00–10:00 (10)	26	26	26
10:00–14:00 (4)	30	36	39
14:00–24:00 (10)	26	26	26
Fluctuation	Yes	Yes	Yes

### RNA Isolation, cDNA Synthesis, and Gene Cloning

TRIzol (Trizol reagent Invitrogen, United States) reagent was used to extract total RNA from sampled insects according to the manufacturer’s instructions. Isolated RNA was stored at −80°C until first-strand cDNA synthesis was performed using a commercial reverse transcription kit (AT341-02, TransGen Biotech, China). Full-length cDNA of *AhHsp70* and *AhsHsp21* of *A. hygrophila* was produced by RT-PCR and rapid amplification of cDNA-ends (RACE)-PCR, and the resulting sequences were submitted to NCBI (GenBank accession numbers: MN138034 and MN163038, respectively, Supplementary Figures S2, S3; Jin et al., 2020a). We used *A. hygrophila* transcriptome data to obtain expressed sequence tags (ESTs) that showed similarities to other insect Hsfs. Polymerase chain reaction (PCR) primers were designed using primer 5.0 (Supplementary Table S2). The PCR reaction was performed following the procedure of Jin et al. (2020a); PCR products were cloned into a pEASY-T3 vector (TransGen, Beijing, China) and were then sequenced.

### Sequence Analysis Identification of *AhHsf* cDNA

The cDNAs of *AhHsf* were used as query sequences to search for other *Hsfs* in GenBank using the BLAST software available at the NCBI website^1^. Sequence identification analyses were carried out using MEGA6 or vector NTI software.

### Relative Quantitative Real Time PCR

Heat shock-regulated gene expression was analyzed using RT-qPCR and TransStart Green qPCR SuperMix Kit with SYBR (Transgen, Beijing, China) with an ABI Prism 7500 Real Time PCR System (Applied Biosystems, NYC, United States). Gene-specific primers for *Hsf* gene amplification by primer 5.0 are shown in Supplementary Table S1. PCR reactions were performed using a total reaction volume of 20 μL comprising 10 μL 2 × *TransStart*^®^ Tip Green qPCR SuperMix (TransGen Biotech, China), 0.4 μL Passive Reference Dye II, 0.4 μL of each of gene-specific primer pair, 1 μL cDNA template, and 7.8 μL ddH_2_O. Each experiment comprised three biological and three technical replicates. Relative expression levels of the target molecule was determined using the C_p_ method according to the mathematical model of Pfaffl (Fleige et al., 2006), using β*-actin* as an internal reference. Aimplified to 2^–△△^^Ct^ as follows:

^△△^ Ct = (C_p_ target-C_p_ reference) _*treatment*__–_(C_p_ target-C_p_ reference) _control_

### Double-Stranded *AhHsf, AhHsp70*, and *AhsHsp21* RNA Synthesis and RNAi

dsRNA was synthesized from the *AhHsf, AhHsp70* and *AhsHsp21* cDNA from *A. hygrophila* using gene-specific primers, and *EGFP* (GenBank Accession No. AIR08541.1) dsRNA was synthesized as a negative control. A T7 promoter as described by Ghanim et al. (2007) was added to the 5′-end of each primer. The promoter sequence was 5′-TAATACGACTCACTATAGGG-3′. PCR products were purified using an AxyPrep^TM^ DNA Gel Extraction Kit (Axygen, Silicon Valley, United States) according to the manufacturer’s instructions. PCR products were stored at −80°C prior to synthesis of dsRNA.

dsRNA was synthesized using the HiScribe^TM^ T7 Quick High Yield RNA Synthesis Kit (New England BioLabs, Ipswich, MA, United States; #E2050S) following the manufacturer’s protocol. The concentration of dsRNA was measured using a NanoVue spectrophotometer (GE Healthcare, Germany), and purity was tested by electrophoresis using a 1.0% agarose gel. To ensure that the injection volume between the control group and treatment group was consistent, the concentration of all synthesized dsRNA was adjusted to 8,000 ng/μL; dsRNA solution (125 nL) was injected in the abdomen of the insects (with a final dsRNA amount of 1.0 μg). Where subsequent experiments were not conducted immediately, dsRNA was stored at −80°C.

### Expression Analysis of *AhHsf* After Injection With *dsAhHsf*

Newly emerged adult *A. hygrophila* females (< 12 h after eclosing) were collected for dsRNA injection using a PLI-100 Pico-Injector (Harvard Apparatus, Holliston, MA, United States) with an MP-255 Micromanipulator (Sutter, Novato, CA, United States) using an Olympus stereomicroscope. ds*AhHsf* solution (125nL; with a final dsRNA amount of 1.0 μg) was injected in the insects’ abdomen. After injection, the insects were kept under standard conditions, as described above. Six adults were collected from each temperature treatment and used for expression analysis of *AhHsf* genes every day for 1 week. The control group individuals were injected with dsRNAs of *EGFP*.

### Knockdown of *AhHsf* Affecting Expression of Two Potential Target *AhHsps*

To confirm the specificity of RNAi, dsRNA solution (the final amount of dsRNA reached 1.0 μg) for *AhHsf* was injected into insects in the penultimate abdomen of newly emerged adult female beetles. Each experiment was performed three times and a total of 50 of adults were used for each temperature treatment. After injection, these beetles were subjected to heat-shock at each of the three temperatures (30, 36, or 39°C) for 4 h from 10:00 to 14:00 in a constant-temperature incubator (RPX-450, NUNON, Beijing) daily. Five females were collected and used for expression analysis of the two *AhHsp* genes by using gene-specific PCR primers at each temperature every day for a week. The blank control was injected with dsRNAs of *EGFP*. β*-actin* gene primers were used as an internal control to monitor equal loading of cDNA for analyses of transcription levels.

### Effects of *dsAhHsp70* or *dsAhsHsp21* Injection on *AhHsf* Expression

dsRNA solution for *AhHsp70, AhsHsp21* or *AhHsp70 and AhsHsp21* was injected into insects in the penultimate abdomen of newly emerged adult female beetles. Each experiment was repeated three times and a total of 50 of adults were used for each treatment. After injection, the expression of *Ahhsf* was analyzed by qRT-PCR. The blank control was injected with ds*EGFP.*

### Effects of RNAi on Fecundity of *A. hygrophila*

After injection with ds*AhHsf*, one newly emerged (< 12 h following eclosion) adult female and one male of *A. hygrophila* were placed together in a Petri dish (with a diameter of 9 cm) containing fresh alligator weed leaves, and they were kept under standard conditions as described above; a total of 15 pairs of adults were used for each temperature treatment and three replicates were performed for each treatment. To ensure the survival of 15 females for later experimental observation after injection, we injected about 50 females for our experiment. The blank control was injected with ds*EGFP*.

### Effects of Injection of dsRNA on *A. hygrophila* Longevity

Newly hatched adult *A. hygrophila* females and males (< 12 h) were collected for ds*AhHsf* injection (30 females and 30 males per treatment). After injection, one female and one male were kept under standard conditions, as described above, for visual monitoring of phenotype changes and further analyses. Individual survival was recorded daily until the last specimen died. The control was injected with ds*EGFP* and replicated three times for each treatment.

### Statistical Analyses

Statistical analyses were performed using SAS software (v8) for Microsoft Windows; data are shown as means ± standard deviation. Experimental data were checked for normality and homoscedasticity, and where needed, were arcsine square-root- or log-transformed before analysis. Target gene expression levels were analyzed using a one-way analysis of variance (ANOVA; SAS Institute Inc., 1996, United States), and a least significant difference test was used to test differences across data. Statistical significance is reported at *p* < 0.05. Genes expression levels and fecundity of *A. hygrophila* after injection with dsRNA were analyzed by Student’s *t*-test. Data on adult survival rates were analyzed using a log-rank (Mantel-Cox) test (Mantel, 1985), and a survival curve was constructed using GraphPad Prism 6 (GraphPad Software Inc., San Diego, CA, United States). When the *p*-values were 0.05 or lower, they were considered significant.

## Results

### Sequence Analysis of *AhHsf*

**FIGURE 1:**
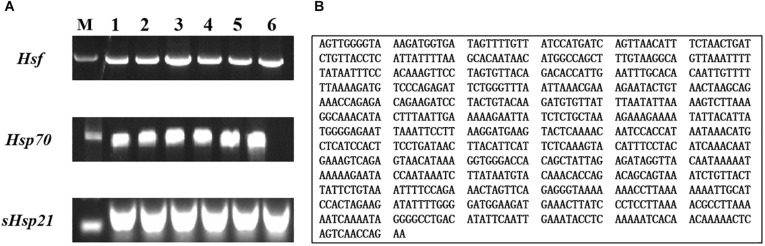
Gel electrophoretogram and nucleotide sequences of *AhHsf* from *A. hygrophila*. **(A)** Gel electrophoretogram; **(B)** Nucleotide sequences of *Hsf*. M: M stands for Trans2K^TM^ DNA marker; 1, 2, 3, 4, 5, and 6 gel lanes represent gel electrophoretogram at different annealing temperatures with the same gene primer. Lane 1, 52°C; Lane 2, 54°C; Lane 3, 56°C; Lane 4, 58°C; Lane 5, 60°C; Lane 6, 62°C; *Hsf*, *Hsp70*, and *sHsp21* respectively represent the gel electrophoretogram of PCR amplification sequences of *AhHsf*, *AhHsp70*, and *AhsHsp21*.

We produced a 792-bp partial cDNA sequence of *AhHsf* by RT-PCR from *A. hygrophila*, which was deposited at the NCBI database (GenBank accession numbers: MT133904) (Figure 1). *AhHsf* sequence analysis indicated that it belongs to the Hsfs gene family (Supplementary Figure S4) by BLAST software available at the NCBI website^1^.

### Expression of the *AhHsf* Gene in *A. hygrophila* Under Heat Stress

The standard curves of the *AhHsf* gene and the housekeeping gene produced a correlation coefficient of 0.998 and 1.000, respectively and the amplification efficiency was 104.852 and 96.265, respectively (Supplementary Figure S5).

**FIGURE 2:**
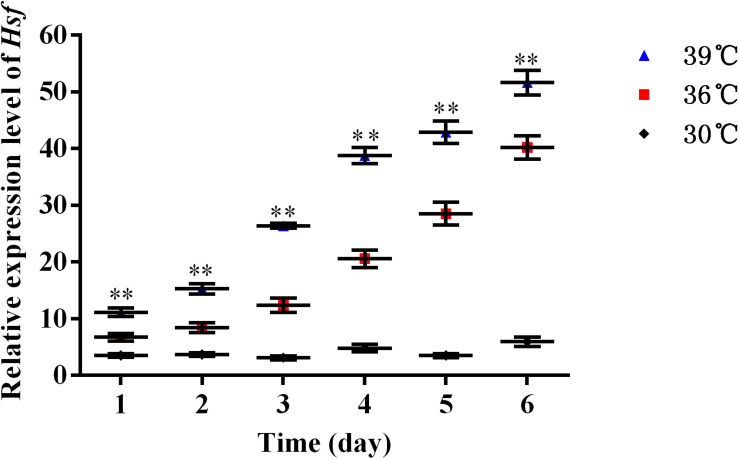
A high temperature of 30, 36, and 39°C induced expression profiles of *AhHsf* in *A. hygrophila*. Relative mRNA levels were analyzed using the 2^–△△Ct^ method. The figure shows data from three replicates that were analyzed by one-way ANOVA followed by the least significant difference (LSD) test. All values are shown as the mean ± SD. ^∗^*P* < 0.05, significant; ^∗∗^*P* < 0.01, extremely significant. ns, not significant.

The expression levels of *AhHsf* mRNAs in *A. hygrophila* under 30, 36, and 39°C temperature treatments were determined using relative quantitative real time PCR. qPCR analysis showed that the expression of the *AhHsf* gene was induced by 4-h heat treatments. Significant up-regulation of *AhHsf* expression was observed when the temperature was further increased from 30 to 39°C. *AhHsf* expression was significantly higher in the 36 and 39°C temperature treatment groups than in the control group kept at 30°C during the 6 days of observation [1d: *F*_(__2_, _8__)_ = 80.60, *p* = 0.0038; 2d: *F*_(__2_, _8__)_ = 188.89, *p* = 0.0013; 3d: *F*_(__2_, _8__)_ = 775.06, *p* = 0.0005; 4d: *F*_(__2_, _8__)_ = 767.06, *p* = 0.0001; 5d: *F*_(__2_, _8__)_ = 565.15, *p* = 0.0009; and 6d: *F*_(__2_, _8__)_ = 491.12, *p* = 0.0001] (Figure 2). The expression of β*-actin* was used as an internal control.

### *AhHsf* and Two *AhHsp* Genes Expression Levels After Injection With *dsAhHsf*

**FIGURE 3:**
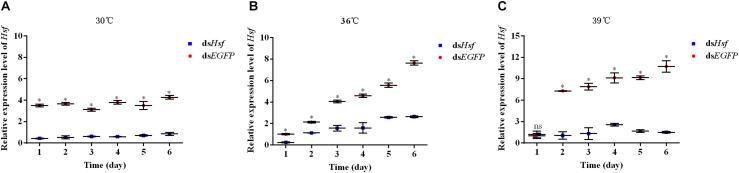
**(A)** Relative expression levels of *AhHsf* after injection of ds*Hsf* under the 30°C condition; **(B)** Relative expression levels of *AhHsf* after injection of ds*Hsf* under the 36°C condition; **(C)** Relative expression levels of *AhHsf* after injection of ds*Hsf* under the 39°C condition. Relative expression levels of *AhHsf* after injection of dsRNA into freshly emerged *A. hygrophila* adults (*n* = 3). Relative mRNA levels were normalized to those of the *CoxI* gene and analyzed using the 2^–△△Ct^ method. All values are shown as the mean ± SD. The data were analyzed by Student’s *t*-test. ^∗^*P* < 0.05. ns, not significant. ds*EGFP* RNA was used as the control.

qPCR assays showed that injection with ds*AhHsf* significantly inhibited endogenous expression of *AhHsf* mRNA at each time point of sampling (1, 2, 3, 4, 5, and 6 days). From 1 to 6 days after injection, the expression level of *AhHsf* mRNA decreased significantly when injected with ds*AhHsf* compared to that when injected with ds*EGFP* at 30°C and 36°C (A: 1d: *t* = 43.29, *p* < 0.05; 2d: *t* = 16.79, *p* < 0.05; 3d: *t* = 35.47, *p* < 0.05; 4d: *t* = 28.68, *p* < 0.05; 5d: *t* = 12.80, *p* < 0.05; and 6d: *t* = 26.54, *p* < 0.05. B: 1d: *t* = 30.61, *p* < 0.05; 2d: *t* = 15.68, *p* < 0.05; 3d: *t* = 18.04, *p* < 0.05; 4d: *t* = 14.41, *p* < 0.05; 5d: *t* = 24.32, *p* < 0.05; and 6d: *t* = 28.10, *p* < 0.05) (Figures 3A,B). At 39°C, from day 2–6, after the injection with ds*AhHsf*, *AhHsf* mRNA level was significantly decreased compared to that in the control group (2d: *t* = 20.10, *p* < 0.05; 3d: *t* = 12.51, *p* < 0.05; 4d: *t* = 15.58, *p* < 0.05; 5d: *t* = 57.62, *p* < 0.05; and 6d: *t* = 19.80, *p* < 0.05) (Figure 3C), However, on the first day, there was no significant difference in the *AhHsf* mRNA expression level between the samples injected with ds*AhHsf* and ds*EGFP* (1d: *t* = −0.72, *p* = 0.5135) (Figure 3C).

**FIGURE 4:**
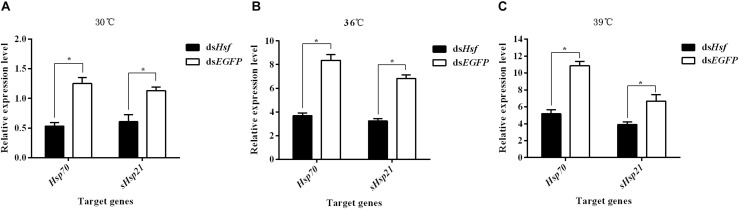
**(A)** Relative expression levels of *AhHsp70*, and *AhsHsp21* after injection of ds*AhHsf* under the 30°C condition; **(B)** Relative expression levels of *AhHsp70*, and *AhsHsp21* after injection of ds*AhHsf* under the 36°C condition; **(C)** Relative expression levels of *AhHsp70*, and *AhsHsp21* after injection of ds*AhHsf* under the 39°C condition. Relative expression levels of *AhHsp70*, and *AhsHsp21* after injection of ds*AhHsf* into freshly emerged female *A. hygrophila* adults. All values are shown as the mean ± SD. The values show data from three replicates that were analyzed using Student’s *t*-test. Different amounts of ds*EGFP* were injected as a control. * *P* < 0.05. ns, not significant.

RT-qPCR analyses of RNA isolated one to 6 days after injection showed that dsRNA suppressed the transcription levels of the target genes *AhHsp70* and *AhsHsp21* (A: *Hsp70*: *t* = −9.06, *p* < 0.05; *sHsp21*: *t* = −6.35, *p* < 0.05. B: *Hsp70*: *t* = −10.41, *p* < 0.05; *sHsp21*: *t* = −14.33, *p* < 0.05. C: *Hsp70*: *t* = −17.68, *p* < 0.05; *sHsp21*: *t* = −5.30, *p* < 0.05) (Figure 4). The results revealed that these two *AhHsps* could be regulated by *AhHsf* at high temperatures.

### Effects of *dsAhHsp70*, *AhsHsp21*, or Their Combination Injection on Expression of *AhHsf* Genes

**FIGURE 5:**
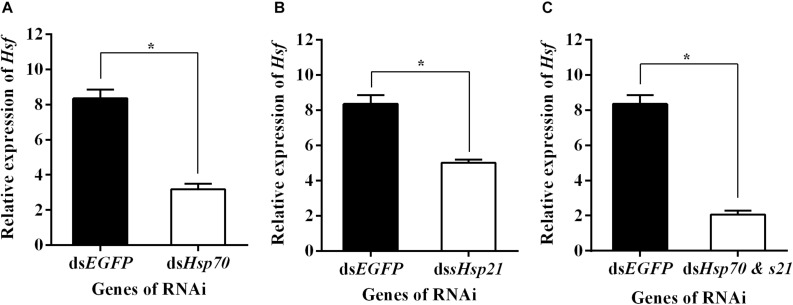
**(A)** Relative expression levels of *AhHsf* after injection of ds*AhHs70*; **(B)** Relative expression levels of *AhHsf* after injection of ds*AhHs21*; **(C)** Relative expression levels of *AhHsf* after injection of ds*AhHs70* and *sHsp21*. Relative expression levels of *AhHsf* after injection of ds*AhHs70*, ds*AhsHsp21*, or ds*AhHs70 and AhsHsp21* into freshly emerged female *A. hygrophila* adults. All values are shown as the mean ± SD. The values show data from three replicates that were analyzed using Student’s *t*-test. Different amounts of ds*EGFP* were injected as a control. * *P* < 0.05. ns, not significant.

qPCR assays showed that *AhHsf* expression in ds*AhHsp70*, ds*AhsHsp21*, or ds*AhHsp70* & *AhsHsp21*- injected samples decreased significantly at sampled compared to that in ds*EGFP* - injected samples (A: *t* = −13.94, *p* < 0.05. B: *t* = −9.14, *p* < 0.05. C: *t* = −16.77, *p* < 0.05) (Figure 5). These results probably suggest that the downstream genes *AhHsp70* and *AhsHsp21*can also influence the expression of upstream gene *AhHsf* through feedback regulation.

### Knockdown of *AhHsf* Affects Fecundity and Survival of *A. hygrophila*

**FIGURE 6:**
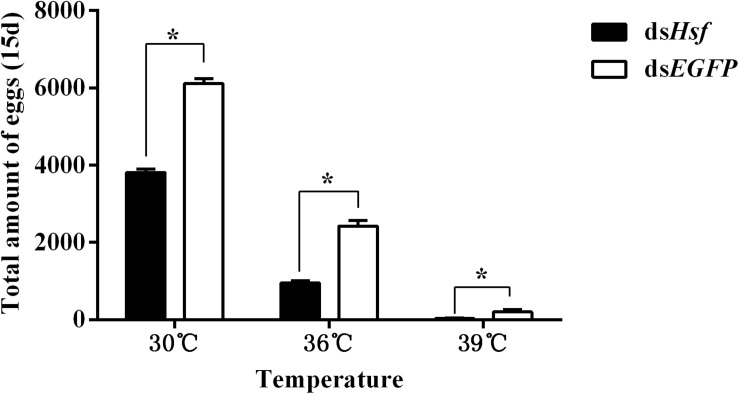
Fecundity of *A. hygrophila* after injection of ds*AhHsf*. Different amounts of ds*EGFP* were injected as a control. A total of 15 pairs of adults were used for each temperature treatment and three replicates were performed for each treatment. All values are shown as the mean ± SD. Data were analyzed using Student’s *t*-test. *P < 0.05. ns, not significant.

The number of eggs produced was significantly lower in individuals injected with ds*AhHsf* than those injected with ds*EGFP* (30°C: *t* = −20.64, *p* < 0.05. 36°C: *t* = −14.63, *p* < 0.05. 39°C: *t* = −3.66, *p* < 0.05) (Figure 6). This decrease was more pronounced in the 39°C treatment group, where female fecundity was close to zero (*t* = −3.66, *p* < 0.05) (Figure 6). Results on fecundity confirmed that RNA interference of *AhHsf* expression significantly inhibits reproduction in *A. hygrophila*.

**FIGURE 7:**
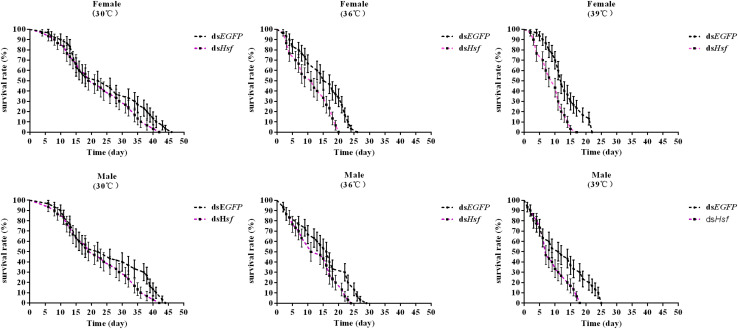
The effect of adult survival of *A. hygrophila* after injection of ds*AhHsf* (*n* = 3). Vertical bars represent standard errors of the mean. Data on the adult survival rate were analyzed using the log-rank (Mantel-Cox) test, and a survival curve was constructed using GraphPad Prism 6.

Silencing of the *AhHsf* gene significantly reduced the survival of *A. hygrophila* in the group in which the temperature was increased, compared to that in the control group where when temperature was 30°C (females −30°C: χ^2^** =** 2.027, *p* = 0.1545; 36°C: χ^2^** =** 9.093, *p* = 0.0026; 39°C: χ^2^** =** 10.65, *p* = 0.0011; males −30°C: χ^2^** =** 2.642, *p* = 0.1041; 36°C: χ^2^** =** 4.551, *p* = 0.0329; 39°C: χ^2^** =** 5.954, *p* = 0.0147) (Figure 7).

## Discussion

Previous studies showed that Hsfs play a central role in remodeling the chromatin structure of Hsps promoter via constitutive interactions with its high-affinity binding site, the HSE (Westwood et al., 1991; Peteranderl and Nelson, 1992; Rabindran et al., 1993; Sarge et al., 1993; Erkine et al., 1999). The interaction between Hsfs and HSE is also critical for stimulating both basal (non-induced) and induced transcription (Erkine et al., 1999). Considering the complexity of the Hsf gene family, knockout of Hsf genes is necessary to determine their respective functional role and biological importance (Kumar et al., 2009). In the present study, injection with ds*AhHsf* significantly decreased the expression of *AhHsp70* and *AhsHsp21* in *A. hygrophila*, compared with the control group that was injected with ds*EGFP*. In addition, the expression of *AhHsf* also decreased significantly after injection with ds*AhHsp70*, ds*AhsHsp21* or ds*AhHsp70* & *AhsHsp21*- injected compared to the control group that was injected with ds*EGFP*. It has been reported in other studies (Sato et al., 1998; Ayté et al., 2001) that downstream genes could also influence the expression of upstream genes through feedback regulation. Ayté et al. (2001) reported that the cell-cycle-regulated transcriptional expression of the cyclin *cig2* gene is dependent on the regulation of the transcription factor Mlu1 cell – cycle box binding factor (MBF) in yeast. However, the deletion of Mlu1 cell – cycle box (MCB) elements in the *cig2* promoter perturbed the expression of not only *cig2* but also of other MBF – dependent genes, indicating that *Cig2p* feedback could regulate the activity of the transcription factor, MBF; therefore, *AhHsf* is involved in the regulation of the heat shock response. This outcome is similar to that reported for *D. melanogaster*, where expression of *Hsf* was affected by heat shock treatment, which produced marked effects on the heat shock response (Sørensen et al., 2007).

In freshly emerged *A. hygrophila* females, injection with ds*AhHsf* significantly reduced expression of the genes *AhHsp70* and *AhsHsp21*, and the treatment also reduced fecundity of *A. hygrophila* female and longevity of adult specimens in general. Our results, which were in corroboration with those of *D. melanogaster*, demonstrated a potential connection that *AhHsf* expression was involved in the up-regulation of *AhHsp70* and *AhsHsp21* in response to high temperatures. Previous studies showed that Nielsen et al. (2005) performed a study on the role of *Hsf* activation for resistance to heat, cold, and high-temperature knock-down and reported that the induction of stress genes was regulated by *Hsf*, such as *Hsps*. In addition, our finding, which showed a correlation between the *AhHsp* and *AhHsf* activities, was consistent with the observations of Edwards et al. (1992), in which a qualitative correlation was seen in humans between levels of *Hsp70* induced in response to heat shock and potentiation by heat shock of progesterone receptor-mediated CAT gene expression; thus, the levels of *Hsp* genes are largely determined by the activity of Hsf. However, the precise mechanism by which increased temperature mediates the AhHsf-AhHsp signaling pathway is not entirely clear and requires further studies.

Previous studies showed that increased temperature elicits activation of a conserved pathway involving heat-shock transcription factor HSF, which enhances the heat shock response (Schuetz et al., 1991; Abravaya et al., 1992; Baler, 1992; Schlesinger and Ryan, 1993; Mosser et al., 1993; Erkine et al., 1999; Singh and Aballay, 2006; Kumar et al., 2009; Gonsalves et al., 2011). Moreover, Li et al. (2019) identified a novel heat shock transcription factor, *REVEILLE 4/8*, in *Arabidopsis* which regulates heat shock-induced gene expression; our study revealed that the heat shock factor in *A. hygrophila* showed a similar expression pattern. The expression levels of *AhHsf* and the two potential target *AhHsps* increased with the increasing heat shock temperature. Therefore, we inferred that the *AhHsf* gene plays an important role in the heat shock response of *A. hygrophila*.

## Conclusion

In this study, we isolated and identified a heat shock factor gene (*AhHsf*) and its two potential downstream genes *AhHsp70* and *AhsHsp21* from *A. hygrophila*. We used RT-qPCR and RNAi technology to detect the effects of *AhHsf* on the expression levels of two potential downstream *AhHsp* genes, fecundity of *A. hygrophila* females, and adult longevity. Our results showed that injection with ds*AhHsf* significantly inhibited the expression of *AhHsp70* and *AhsHsp21* mRNAs and decreased fecundity of *A hygrophila* female and adult longevity. Our qPCR assays showed that *AhHsf* expression in ds*AhHsp70*, ds*AhsHsp21*, or ds*AhHsp70* & *AhsHsp21*- injected samples decreased significantly compared to that in ds*EGFP*-injected samples. Therefore, our findings provide evidence that *AhHsf* is involved in regulating the transcriptional expression of *AhHsp70* and *AhsHsp21* and plays a key role in thermotolerance in *A. hygrophila*. These results may improve our understanding of the molecular mechanisms of the AhHsf- AhHsp signaling pathways in *A. hygrophila.*

## Data Availability Statement

The datasets generated for this study can be found in online repositories. The names of the repository/repositories and accession number(s) can be found in the article/ Supplementary Material.

## Author Contributions

JJ, JG, ZZ, and FW conceived and designed the experiments. JJ conducted the experiments, bioinformatic analyses, RT-PCR, qPCR, and RNAi. JJ, ZZ, and YL contributed to data analyses. JJ wrote the manuscript. All authors contributed to the article and approved the submitted version.

## Conflict of Interest

The authors declare that the research was conducted in the absence of any commercial or financial relationships that could be construed as a potential conflict of interest.

## Footnotes

1 https://blast.ncbi.nlm.nih.gov/Blast.cgi
