# Supplementary material for: Heat Shock Factor Is Involved in Regulating the Transcriptional Expression of Two Potential Hsps (AhHsp70 and AhsHsp21) and Its Role in Heat Shock Response of Agasicles hygrophila
Source: Front Physiol. 2020 Sep 15;11:562204. doi: 10.3389/fphys.2020.562204 (PMC7522579; doi:10.3389/fphys.2020.562204)
Supplement: Supplementary file 1 [file Table_1.DOCX]

**Supplementary figures**


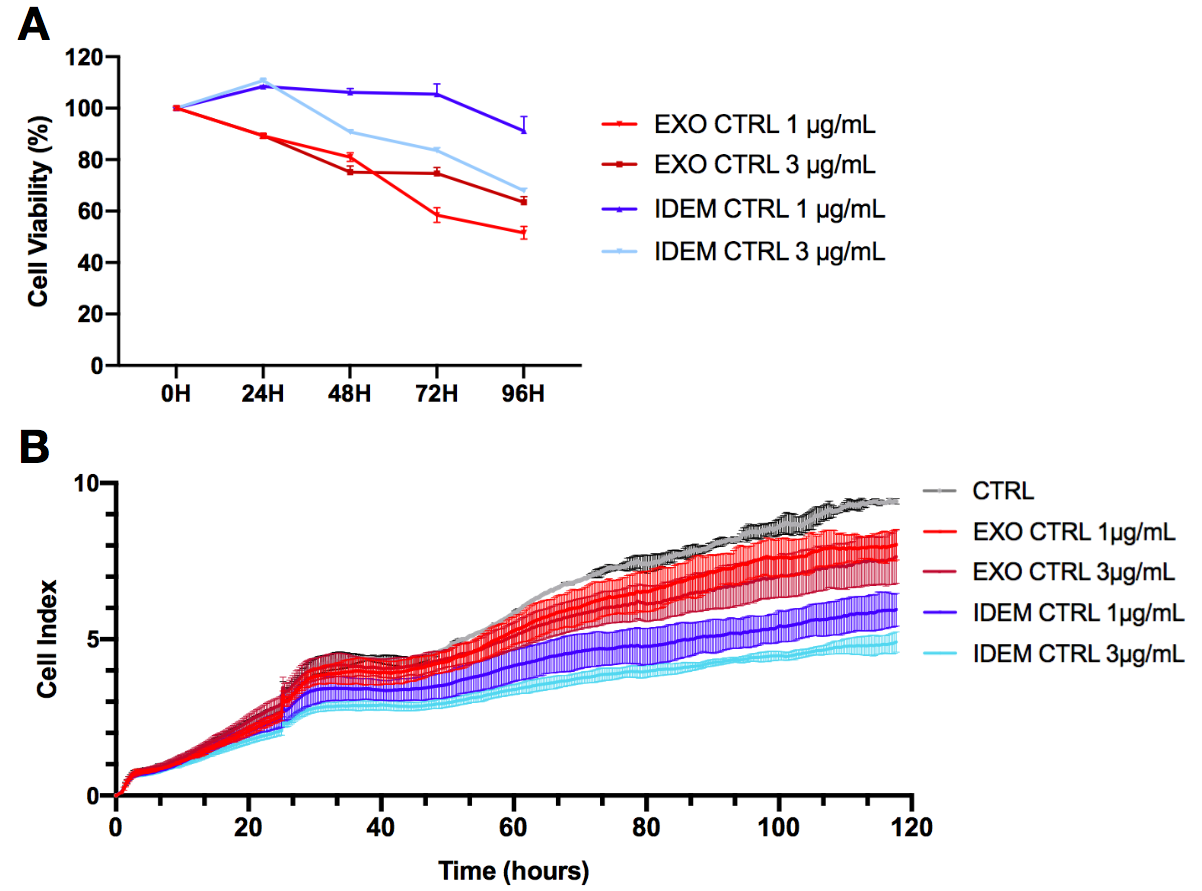


**Figure S1. Cell viability assessment of empty nanoparticles.** (**A**) Alamar blue analysis of SKOV-3 cells treated with different doses (1 and 3 μg/mL) of empty IDEM (IDEM CTRL) and EXO (EXO CTRL) over 96 h. Data show average and standard deviation for all values. Values were normalized to the untreated control. (**B**) xCELLigence cell index data obtained treated SKOV-3 cells with empty IDEM and EXO, which confirm both formulations are not toxic for the cells. Data show average and standard deviation values for each time point (n=3).

**
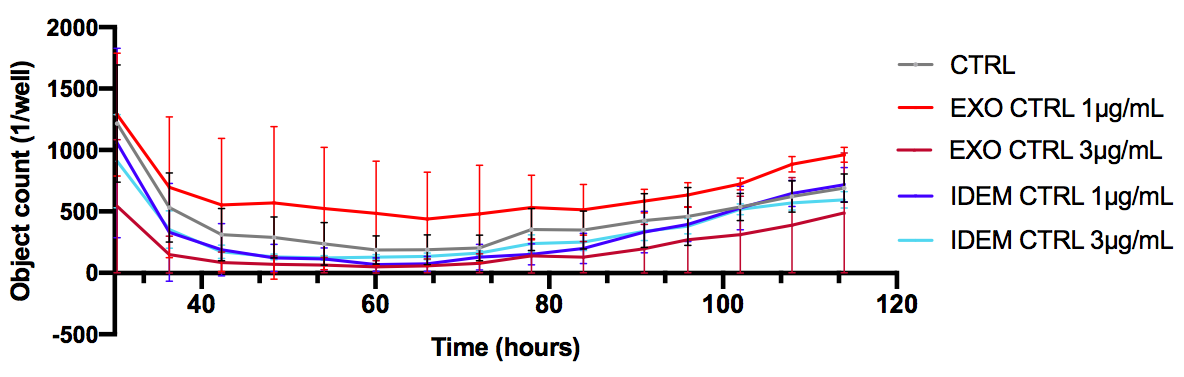
Figure S2. Apoptosis assessment of empty nanoparticles.** Both IDEM and EXO empty particles (EXO CTRL and IDEM CTRL, respectively) were tested at different concentrations (1 and 3 μg/mL) to confirm that they do not induce caspase3-dependent apoptosis on SKOV-3 cells. All treatments follow the same trend as the untreated SKOV-3 cells (CTRL). Data are expressed as average with standard deviation for each time point (n=3).
